# SnRK1 phosphorylation of FUSCA3 positively regulates embryogenesis, seed yield, and plant growth at high temperature in Arabidopsis

**DOI:** 10.1093/jxb/erx233

**Published:** 2017-07-22

**Authors:** Aaron Chan, Carina Carianopol, Allen Yi-Lun Tsai, Kresanth Varatharajah, Rex Shun Chiu, Sonia Gazzarrini

**Affiliations:** 1 Department of Biological Sciences, University of Toronto Scarborough, Military Trail, Toronto, ON Canada; 2 Department of Cell and Systems Biology, University of Toronto, Toronto, ON Canada

**Keywords:** AKIN10, embryogenesis, FUS3, heat stress, high temperature, phosphorylation, seed development, SnRK1, transcriptional regulation

## Abstract

The transcription factor FUSCA3 (FUS3) acts as a major regulator of seed maturation in Arabidopsis. FUS3 is phosphorylated by the SnRK1 catalytic subunit AKIN10/SnRK1α1, which belongs to a conserved eukaryotic kinase complex involved in energy homeostasis. Here we show that AKIN10 and FUS3 share overlapping expression patterns during embryogenesis, and that FUS3 is phosphorylated by AKIN10 in embryo cell extracts. To understand the role of FUS3 phosphorylation, we generated *fus3-3* plants carrying FUS3 phosphorylation-null (FUS3^S>A^) and phosphorylation-mimic (FUS3^S>D^) variants. While FUS3^S>A^ and FUS3^S>D^ rescued all the *fus3-3* seed maturation defects, FUS3^S>A^ showed reduced transcriptional activity and enhanced *fus3-3* previously uncharacterized phenotypes. FUS3^S>A^ embryos displayed increased seed abortion due to maternal FUS3^S>A^ and delayed embryo development, which correlated with a strong decrease in seed yield (~50%). Accordingly, the *akin10* and *akin11* mutants displayed a frequency of seed abortion similar to *fus3-3*. When plants were grown at elevated temperature, most phenotypes were exaggerated in FUS3^S>A^ plants, and progeny seedlings overall grew poorly, suggesting that phosphorylation of FUS3 plays an important role during early embryogenesis and under heat stress. Collectively, these results suggest that FUS3 phosphorylation and SnRK1 are required for embryogenesis and integration of environmental cues to ensure the survival of the progeny.

## Teaser

FUS3 phosphorylation at the SnRK1 target site regulates embryo growth rate, seed yield, and plant growth at control and elevated temperature.

## Introduction

Seed production is vital to the success of higher plants and integrally important to human diet and animal feed. Seed development is a highly regulated and complex process. *Arabidopsis thaliana* follows a classical pattern of double fertilization forming the zygote and endosperm. Cell division dominates early embryogenesis up until the globular stage; afterwards cell differentiation overtakes division in mid to late embryogenesis. In conjunction, seed storage reserves accumulate, dormancy is established, and desiccation tolerance is acquired, resulting in the final mature seed (Capron *et al.*, 2009).

The LAFL proteins, LEAFY COTYLEDON 2 (LEC2), ABSCISIC ACID INSENSTIVE 3 (ABI3), and FUSCA3 (FUS3) B3 domain transcription factors, together with LEC1 and LEC1-LIKE (L1L), homologs of the NF-YB subunit of the CCAAT-binding complex, are considered master regulators of seed development. Mutations in these genes cause reduced levels of seed storage compounds, desiccation intolerance, and/or enhanced precocious germination of immature embryos, indicating their critical role in seed maturation (Jia *et al.*, 2013; Nonogaki, 2014; Fatihi *et al.*, 2016). Mutant analyses suggest that each gene acts during different developmental stages of seed maturation to arrest embryo growth, with *fus3-3* mutants acting earliest and showing premature growth of excised embryos, from the torpedo stage onwards (Raz *et al.*, 2001). Almost complete repression of somatic embryogenic potential is seen in *lec1/2* and *fus3* mutants, further demonstrating the requirement of these genes for the embryonic program (Gaj *et al.*, 2005). In accordance, in ChIP using somatic embryo tissue, FUS3 was bound to seed-specific promoters, with the majority containing the FUS3/LEC2/ABI3 target RY *cis*-motif (Reidt *et al.*, 2000; Wang and Perry, 2013). Transcription of some of these seed-specific genes, such as those encoding storage proteins, *CRUCIFERIN 3* (*CRU3*), *LATE EMBRYOGENESIS ABUNDANT* (*LEA*), and *SEED STORAGE ALBUMIN 3* (*2S3*), have been demonstrated to be regulated by both *FUS3* and *ABI3* (Parcy *et al.*, 1997; Nambara *et al.*, 2000; Kagaya *et al.*, 2005; Yamamoto *et al.*, 2010; Roscoe *et al.*, 2015).

Restriction of *AFL* function to seed development has been shown to be essential for the transition to vegetative growth and to be under epigenetic regulation (Jia *et al.*, 2013; Fatihi *et al.*, 2016). Ectopic expression of *FUS3* during vegetative growth up-regulates seed-specific gene transcription, delays germination, growth, and flowering, and causes embryonic-like leaf development (Gazzarrini *et al.*, 2004; Kagaya *et al.*, 2005; Tsai and Gazzarrini, 2012*b*). Embryonic phenotypes are also shown in plants overexpressing the *LEC* genes (Lotan *et al.*, 1998; Stone *et al.*, 2001). Accordingly, in higher order *viviparous1/abi3-like*- (*val*), *curly leaf*- (*clf*), and *pickle*- (*pkl*) related mutants involved in chromatin remodeling, failure to suppress *FUS3/LEC2/ABI3* expression during germination results in expression of embryonic traits and developmental arrest of seedlings (Jia *et al.*, 2013; Fatihi *et al.*, 2016).

Genetic, transcriptomic, and ChIP studies have shown that spatio-temporal expression of FUS3/LEC2/ABI3 is transcriptionally cross-regulated during seed development (Parcy *et al.*, 1997; To *et al.*, 2006; Mönke *et al.*, 2012; Wang and Perry, 2013). Although extensive work has shown the importance of these genes in transcriptional regulation, little is known about how these proteins are themselves regulated during seed development. *LAFL* genes interact with hormone signaling and synthesis pathways at various levels to regulate seed development and inhibit germination (Jia *et al.*, 2013). FUS3 in particular is a node in hormone crosstalk and acts as a molecular switch in the dormancy to germination transition (Gazzarrini and Tsai, 2015). FUS3 inhibits the synthesis of gibberellic acid (GA) and ethylene, and promotes that of abscisic acid (ABA) to induce dormancy while inhibiting germination and vegetative growth (Curaba *et al.*, 2004; Gazzarrini *et al.*, 2004; Lumba *et al.*, 2012). ABA and GA positively and negatively regulate the FUS3 protein through a PEST degron (sequence rich in proline, glutamic acid, serine, and threonine), respectively (Gazzarrini *et al.*, 2004; Lu *et al.*, 2010). The FUS3 protein, which is not detected during germination at optimal temperature, accumulates during seed imbibition at high temperature due to an increase in the ABA/GA ratio, which inhibits FUS3 degradation through inhibition of proteasome activity. Seeds overexpressing FUS3 are hypersensitive to high temperature and inhibit germination through *de novo* ABA synthesis (Chiu *et al.*, 2012, 2016*a*, *b*). This indicates that FUS3 accumulation is under tight hormonal control and regulates both primary and secondary dormancy.

Snf1-related kinase1 (SnRK1) is a conserved eukaryotic kinase complex involved in cellular energy homeostasis. In Arabidopsis, there are three homologs of the kinase α-subunit, of which AKIN10/SnRK1α1 and AKIN11/SnRK1α2 are functional (Crozet *et al.*, 2014). Single mutants of SnRK1α show a slight delay in flowering and lower transcript levels of darkness-induced genes, while a virally induced *akin10/akin11* RNAi double mutant undergoes premature senescence and is non-viable. SnRK1 overexpression elicits transcriptome shifts to inhibit growth and promote survival (Baena-González *et al.*, 2007; Mair *et al.*, 2015; Nukarinen *et al.*, 2016). In peas (*Pisum sativum*), an antisense *SnRK1α* construct driven by a seed-specific promoter results in enhancement of vivipary, reduced desiccation tolerance, and lower levels of seed storage compounds and ABA, similar to *fus3/lec1/lec2/abi3* mutants, and correspondingly causes reduced transcript levels of *FUS3* and *LEC1* orthologs. SnRK1 knockdown lines also displayed seed abortion and delayed embryo development, suggesting that SnRK1 plays an important role during seed development (Radchuk *et al.*, 2006, 2010).

FUS3 is phosphorylated by AKIN10/SnRK1α1 (Tsai and Gazzarrini, 2012*a*). Overexpression of AKIN10 delays germination, senescence, and flowering (Baena-González *et al.*, 2007; Tsai and Gazzarrini 2012*a*, *b*; Jeong *et al.*, 2015), which can be rescued by *fus3-3*, indicating that *FUS3* acts downstream of *AKIN10* (Tsai and Gazzarrini, 2012*a*). Although overexpression of AKIN10 delays FUS3 degradation, the role of FUS3 phosphorylation by AKIN10 in seeds is unknown (Tsai and Gazzarrini, 2012*a*). In this study, we examined the function of the SnRK1 phosphorylation sites of FUS3 (S55, S56, S57) through analysis of FUS3 phosphorylation-null (FUS3^S>A^) and phosphorylation-mimic (FUS3^S>D^) mutants. While the FUS3^S>D^ variant rescued *fus3-3* seed maturation defects, FUS3^S>A^ showed increased seed abortion, an increased number of seedling showing polycotyledons [previously observed in *fus3*-3 (Tsai and Gazzarrini, 2012*a*)], delayed embryo development, and reduced seed yield. Interestingly, *akin10* and *akin11* mutants displayed a frequency of seed abortion similar to *fus3-3*. Furthermore, when *fus3-3* and FUS3^S>A^ complemented lines were grown under elevated temperature, they displayed reduced plant vigor, seed yield, and seedling growth of the progeny. These results uncover a new function for FUS3 phosphorylation during embryogenesis and under heat stress.

## Materials and methods

### Plant material, growth conditions, and phenotypic analysis

The AKIN10–green fluorescent protein (GFP) transgenic line, and *akin10* and *akin11* mutants were previously described (Bitrián *et al.*, 2011; Tsai and Gazzarrini, 2012*a*; Mair *et al.*, 2015). FUS3 phosphomutants were generated by site-directed mutagenesis of a previously described pFUS3:FUS3-eGFP/pBI construct (Gazzarrini *et al.*, 2004) using QuikChange^®^ (Agilent). The non-phosphorylatable or phospho-null (*pFUS3:FUS3*^*S>A*^*-GFP*) and phosphorylation-mimic or phospho-mimic (*pFUS3:FUS3*^*S>D*^*-GFP*) transgenes were then introduced into the Arabidopsis Columbia *fus3-3* mutant (Keith *et al.*, 1994) by *Agrobacterium*-mediated plant transformation. Several transgenic plants were selected, and experiments were conducted using two independent homozygous lines. Arabidopsis seeds were germinated on half-strength Murashige and Skoog (MS) medium, transferred to soil at 7 d after germination, and grown under long days at 16 h light 21 °C/8 h dark 18 °C (optimal temperature regime) or 16 h light 27 °C/8 h dark 24 °C (high temperature regime).

For germination and seedling establishment assays, three sets of 50 seeds were cold stratified and germinated for 7 d. Bolting time was counted when the primary inflorescence reached 2 cm in length. An average of 15–20 plants per genotype was used. Experiments were repeated twice, and one is shown. Total seed production per silique and seed abortion were determined by counting the number of funiculi and seeds in eight siliques per genotype. Embryo development was scored by the morphological shape of dissected embryos from four siliques per genotype. Growth arrest was scored by growth of dissected embryos on 0.4% water agar after 5 d under constant light at 22 °C. Seed yield was measured from the total weight of dry seeds of eight plants per genotype grown in two pots.

### Microscopy

Light microscopy images were taken using an SMZ1500 dissecting microscope (Nikon). Confocal images were taken using an LSM510 (Zeiss) with a 488 nm excitation laser with a 515–535 nm bandpass filter for GFP emission (green channel) and a 595 nm high-pass filter (red channel). Plant tissue was directly mounted on glass slides in 10% glycerol.

### Protein expression analysis

Proteins were extracted by grinding frozen plant material in 2× Laemmli buffer (120 mM Tris pH 6.8, 20% glycerol, 4% SDS, 10% β-mercaptoethanol, 0.004% bromophenol blue) followed by 10 min of boiling and separation of debris by centrifugation. AKIN10 protein was detected using an AKIN10 antibody (Agrisera).

### In-gel kinase activity assay

The in-gel kinase activity assay was performed as previously described (Tsai and Gazzarrini, 2012*a*). Six to ten frozen siliques were homogenized in extraction buffer (100 mM HEPES pH 7.5, 5 mM EDTA, 5 mM EGTA, 10 mM Na_3_VO_4_, 10 mM NaF,50 mM β-glycerophosphate, 10 mM DTT, 1 mM phenylmethylsulfonyl fluoride,1× protease inhibitor, 5% glycerol), and centrifuged twice. A 15 µg aliquot of cell lysate was separated on a 10% SDS–polyacrylamide gel containing 0.1 mg ml^–1^ glutathione *S*-transferase (GST)–FUS3. SDS was removed by washing the gel three times for 30 min at room temperature in washing buffer (25 mM Tris–HCl pH 7.5,0.5 mM DTT, 0.1 mM Na_3_VO_4_, 5 mM NaF, 0.5 mg ml^–1^ BSA, 0.1% Triton X-100). Proteins were renatured by three washes with renaturation buffer overnight (25 mM Tris–HCl, pH 7.5,1 mM DTT, 0.1 mM Na_3_VO_4_, 5 mM NaF). The gel was incubated in reaction buffer for 30 min (25 mM HEPES pH 7.5 2 mM EGTA, 12 mM MgCl_2_, 1 mM DTT,0.1 mM Na_3_VO_4_), then in 30 ml of reaction buffer with 200 nM ATP and 50 μCi of [γ-^32^P]ATP. The reaction was quenched in 5% trichloroacetic acid (w/v) and 1% sodium pyrophosphate (w/v) solution, washed five times to remove any unincorporated radioactivity, and then subjected to autoradiography.

### Gene expression analysis

RNA was extracted from ground frozen plant material using the RNeasy Plant Mini Kit (Qiagen), followed by reverse transcription using GoScript™ (Promega). Quantative PCR was performed using SsoFast™ EvaGreen^®^ (BioRad) with the CFX Connect™ real-time PCR detection system (BioRad). Primers used are listed in Supplementary Table S1 at *JXB* online.

## Results

### AKIN10 is expressed during embryogenesis and phosphorylates FUS3

**Fig. 1.**
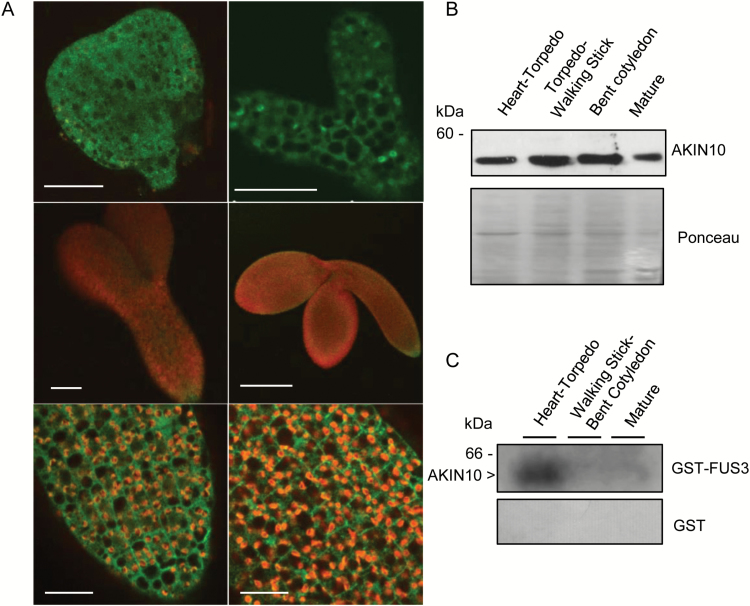
AKIN10 is expressed during embryogenesis and phosphorylates FUS3. (A) Expression pattern of AKIN10 during seed development in Arabidopsis grown under long days (21 °C/18 °C). Confocal images showing AKIN10–GFP expression predominantly in the cytoplasm and occasionally in the nuclei of the embryo proper in the triangular (top, left), heart (top, right), torpedo (center and bottom, left), and bent cotyledon (center and bottom, right) stage embryos. Bottom images are magnifications of torpedo (left) and bent cotyledon (right) embryos shown in the center panels. Scale bars from left: top, 20 µm; middle, 50 µm, 200 µm; bottom, 20 µm. (B) AKIN10 protein was detected throughout seed development from total soluble protein of isolated seeds using anti-AKIN10. Ponceau stain is shown as the loading control. (C) In-gel kinase assay using purified GST–FUS3 (top) or GST (bottom) as the substrates and cell extracts from siliques showing a phosphorylation band at the expected size of AKIN10 at the heart/torpedo stages. Purified GST was used as the negative control. (This figure is available in colour at *JXB* online.)

It has been previously shown that FUS3 is phosphorylated by AKIN10 on serine residues 55, 56, and 57 in an in-gel kinase assay using cell extract from seedlings (Tsai and Gazzarrini, 2012*a*). However, the FUS3 protein has only been detected during embryogenesis using a FUS3–GFP reporter, although mRNA is also found post-embryonically (Gazzarrini *et al.*, 2004; Lu *et al.*, 2010; Tsai and Gazzarrini, 2012*a*). To test whether AKIN10 may phosphorylate FUS3 *in vivo*, we first examined the expression pattern of AKIN10 during seed development. In transcriptomic studies, *FUS3* and *AKIN10* transcripts have been detected throughout embryo development, and as early as the elongated zygote stage (Le *et al.*, 2010; Xiang *et al.*, 2011). Using the AKIN10–GFP line previously described (Bitrián *et al.*, 2011), the AKIN10 protein was found to be expressed in all cells from the heart to mature embryo stages, which was also confirmed by western blots (Fig. 1A, B; Supplementary Fig. S1). Thus, the AKIN10–GFP expression pattern during embryogenesis overlaps with that of FUS3–GFP previously published (Gazzarrini *et al.*, 2004). Furthermore, in-gel kinase assays show that AKIN10 from cell extracts of siliques corresponding to early embryo stages can phosphorylate FUS3 (Fig. 1C; Supplementary Fig. S2), suggesting that AKIN10 interaction with and phosphorylation of FUS3 is biologically relevant and may play a role during early to mid embryogenesis.

### FUS3 phosphorylation at SnRK1 sites positively regulates expression of FUS3 target genes

**Fig. 2.**
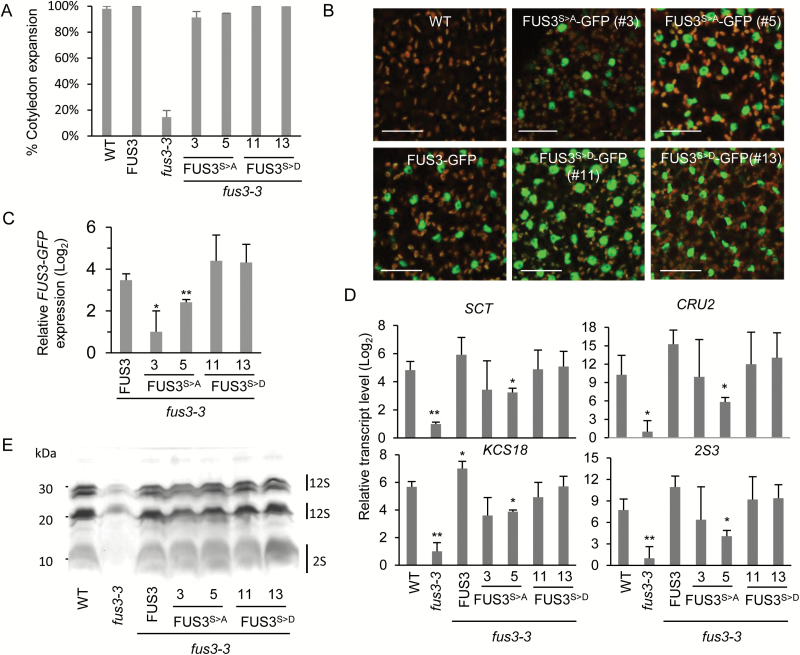
Expression levels of *FUS3* and FUS3 target genes in FUS3 phosphomutants. (A) Seedling establishment (cotyledon expansion) assay of 3-month-old seeds showing rescue of *fus3-3* desiccation intolerance by FUS3:FUS3-GFP, FUS3:FUS3^S>A^-GFP (lines #3 and #5), and FUS3:FUS3^S>D^-GFP (lines #11 and #13) phosphomutant lines. Seeds were cold stratified for 3 d and grown under constant light at 22 °C. The averages of three plates of 50 seeds ±SD are shown. (B) FUS3^S>A^–GFP and FUS3^S>D^–GFP were detected in the nuclei of almost all embryo proper cells in the cotyledon of walking stick/bent embryos. Scale bar=20 µm. (C) qPCR showing decreased *FUS3*^*S>A*^-*GFP* and slightly increased *FUS3*^*S>D*^-*GFP* transcript levels in transgenic lines. All plants were grown under long days (21 °C/18 °C) and siliques were collected at ~10 DAP (walking stick/bent cotyledon stages). (D) Transcript levels of FUS3 target genes measured by qPCR. 12S storage protein *CRU2* (At1G03880), 2S storage protein *2S3* (At4G27160), fatty acid elongase *FAE1*/*KCS18* (AT4G34520), and scorpion toxin proteinase/trypsin inhibitor *SCT* (At1g47540). FUS3^S>A^ lines show lower transcript levels of most genes. Averages of three biological replicates ±SD are shown. Statistical significance against the wild type (WT) calculated by Welch’s *t*-test (*P<0.05, **P<0.01). All plants were grown under long days (21 °C/18 °C) and siliques were collected at ~10 DAP (walking stick/bent cotyledon stages). (E) Seed storage proteins extracted from 1 mg of dry seed visualized by Coomassie staining on a 15% SDS–polyacrylamide gel electrophoresis. (This figure is available in colour at *JXB* online.)

To elucidate the function of FUS3 phosphorylation by SnRK1, we mutated serine residues 55, 56, and 57 of the *pFUS3:FUS3-GFP* construct (Gazzarrini *et al.*, 2004) to alanine and aspartic acid to generate phosphorylation-null (*FUS3*^*S>A*^) and potential phosphorylation-mimic (*FUS3*^*S>D*^) constructs driven by the *FUS3* promoter, respectively. Substitution of serine with aspartic acid has been shown to mimic phosphorylation in several proteins including transcription factors (Maréchal *et al.*, 1999; Kawamoto *et al.*, 2015; Zorzatto *et al.*, 2015). Both constructs were introduced into the *fus3-3* background and rescued the desiccation intolerance phenotype of *fus3-3* seeds (Fig. 2A). In the embryo, FUS3^S>A^–GFP and FUS3^S>D^–GFP were predominately localized to the nucleus, similar to wild-type FUS3–GFP, indicating that phosphorylation was not required for FUS3 subcellular localization and both mutant proteins were functional (Fig. 2B).

Interestingly, fewer FUS3^S>A^ lines were recovered compared with FUS3^S>D^, suggesting that phospho-null mutations provide weaker rescue. This may be due to lower mRNA levels of *FUS3*^*S>A*^ compared with *FUS3* and *FUS3*^*S>D*^ transgenes (Fig. 2C). Given that FUS3 binds to its own promoter and increases its own expression (Parcy *et al.*, 1997; To *et al.*, 2006), these data suggest that FUS3 transcriptional activity may be increased by phosphorylation. To test this hypothesis, we investigated FUS3 transcriptional target gene expression. ChIP studies have shown that FUS3 binds to the promoters of genes that are highly expressed during seed maturation (Wang and Perry 2013), including those encoding 12S storage protein *CRU2* (At1G03880), 2S storage protein *2S3* (At4G27160), fatty acid elongase *FAE1*/*KCS18* (AT4G34520), and scorpion toxin proteinase/trypsin inhibitor *SCT* (At1g47540). These genes are expressed throughout embryogenesis (Toufighi *et al.*, 2005) and their transcript levels were strongly reduced in *fus3-3*; thus, they were good markers for FUS3 transcriptional activity (Fig. 2D; Yamamoto *et al.*, 2010). Transcript measurements taken at the bent cotyledon stage [10 days after pollination (DAP)] show that the FUS3^S>A^ line has lower transcript levels of these genes compared with the FUS3 and FUS3^S>D^ lines (Fig. 2D). However, the levels of storage proteins in dry seeds did not appear to be affected in FUS3^S>A^ lines (Fig. 2E). Thus, although phosphorylation modulates FUS3 transcriptional activity, it did not appear to be required for seed maturation processes such as desiccation tolerance and storage protein accumulation under normal growth conditions.

### FUS3 phosphorylation is required for early embryo development and positively impacts seed yield

**Fig. 3.**
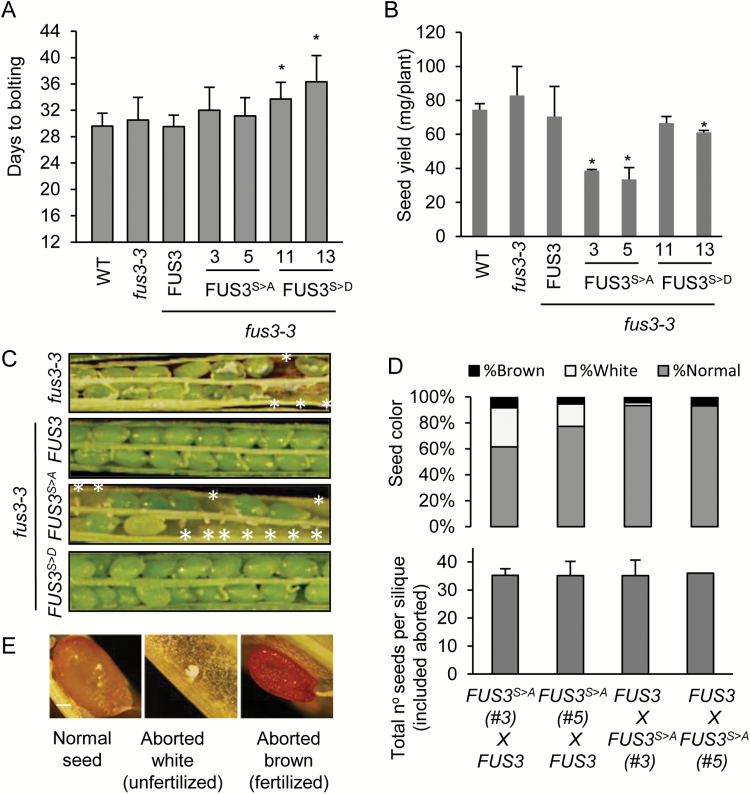
Increased seed abortion and reduced seed yield in FUS3 phospho-null mutants. (A) Bolting time of wild-type, *fus3-3,* FUS3^S>A^, and FUS3^S>D^ plants. Averages of 15–20 plants ±SEM (*P*<0.01) per genotype are shown. Arabidopsis plants were grown under long days at 21 °C/18 °C. (B) Seed yield of FUS3^S>A^ and FUS3^S>D^ plants grown under long days at 21 °C/18 °C. Averages ±SD of total dry seed yield from eight plants grown in two pots is shown (*P*<0.01). (C) Representative picture showing seed morphology in yellowing siliques. *fus3-3* and FUS3^S>A^ show aborted seeds (empty spaces indicated by*). (D) Frequency of seed abortion in F_1_ progeny from reciprocal crosses (♀ × ♂) between FUS3 and FUS3^S>A^. The total number of seeds per silique (bottom) and frequency of aborted white or brown seeds are shown (top). (E) Representative picture showing normal, aborted white (unfertilized), and brown (fertilized) seeds. (This figure is available in colour at *JXB* online.)

Previous work has shown that silencing and overexpression of *AKIN10* cause dramatic phenotypes during vegetative and reproductive development, including alteration of growth rate, senescence, and fertility (Baena-González *et al.*, 2007; Tsai and Gazzarrini, 2012*a*, *b*). This suggests that phosphorylation of FUS3 by SnRK1 could affect vegetative and reproductive development. When grown under long days at 21 °C/18 °C, FUS3^S>D^ lines consistently displayed a delay in flowering (Fig. 3A), similar to plants overexpressing FUS3 and AKIN10 (Gazzarrini *et al.*, 2004; Baena-González *et al.*, 2007; Lu *et al.*, 2010; Tsai and Gazzarrini, 2012*b*). Given that neither FUS3 nor FUS3-phosphomutant proteins were expressed during vegetative development (Supplementary Fig. S3), embryonic FUS3^S>D^ could affect the regulation of genes that control flowering time and are also expressed in the embryo, such as the floral repressor FLOWERING LOCUS C or other regulators of flowering that are expressed in the embryo (Sheldon *et al.*, 2008).

In contrast, FUS3^S>A^ plants appeared less vigorous and their seed yield was reduced by ~50% compared with the wild type and *fus3-3* (Fig. 3B). To understand the cause of decreased seed yield of the FUS3^S>A^ plants, the seed set per silique and quality of seeds produced were examined. When grown under long days, 25–35% of the FUS3^S>A^ seeds were aborted (Fig. 3C; Table 1). Aborted seeds were randomly distributed within a silique and among siliques. Surprisingly, seed abortion was also displayed by *fus3-3*, but at a much lower frequency, ~8% (Table 1). To test if the higher number of aborted seeds of FUS3^S>A^ was due to a zygotic or gametic effect, we crossed FUS3^S>A^ with FUS3 and analyzed the F_1_ progeny of reciprocal crosses. When FUS3^S>A^ was the pollen donor, seed abortion was comparable with *fus3-3*, at 7%, indicating that maternal FUS3 and paternal FUS3^S>A^ did not rescue the *fus3-3* seed abortion phenotype. However, in the reciprocal cross with maternal FUS3 ^S>A^ and paternal FUS3, seed abortion was increased to 23–39%, with a higher frequency of white aborted seeds due to unfertilized ovules (Fig. 3D, E; Table 2). These results show that the lower seed yield of FUS3^S>A^ is due in part to an increase in seed abortion due to a maternal effect of FUS3^S>A^. Lastly, *akin10* and *akin11* mutants showed a rate of seed abortion similar to that of *fus3-3* (Table 1), suggesting that FUS3 and AKIN10 may interact to regulate early embryo development processes.

**Table 1.** Frequency of aborted seeds in various genotypes grown at optimal temperature

Genotype	Total no. of seeds	Total no. of aborted seeds	% Aborted seeds
Wild type	398	8	2%
*fus3-3*	342	26	8%
*fus3-3, FUS3*	381	7	2%
*fus3-3, FUS3* ^*S>A*^ *(3*)	284	100	35%
*fus3-3, FUS3* ^*S>A*^ *(5*)	327	81	25%
*fus3-3, FUS3* ^*S>D*^ *(11*)	383	23	6%
*fus3-3, FUS3* ^*S>D*^ *(13*)	396	8	2%
*akin10*	381	44	12%
*akin11*	402	39	10%

Total seeds produced from eight siliques including aborted seeds were counted.

Seed abortion included both fertilized and unfertilized ovules.

Plants were grown under long days at 21 °C/18 °C.

**Table 2.** Frequency of aborted seeds in progeny from F_1_ crosses

Crosses (♀×♂)	Total no. of seeds	White aborted seeds	Brown aborted seeds	Total no. of aborted seeds
*FUS3* ^*S>A*^ *(3)×FUS3*	179	54 (30%)	15 (8%)	69 (39%)
*FUS3* ^*S>A*^ *(5)×FUS3*	314	54 (17%)	17 (5%)	61 (23%)
*FUS3×FUS3* ^*S>A*^ *(3*)	210	5 (2%)	9 (4%)	14 (7%)
*FUS3×FUS3* ^*S>A*^ *(5*)	72	0 (0%)	5 (7%)	5 (7%)

Total seeds produced including aborted seeds from crossed plants were counted after mature seeds began browning.

Aborted seeds were classified as unfertilized ovules (white) and aborted embryos (brown) based on coat color and morphology.

Plants were grown under long days at 21 °C /18 °C.

### FUS3 phosphorylation positively regulates embryo growth rate

**Fig. 4.**
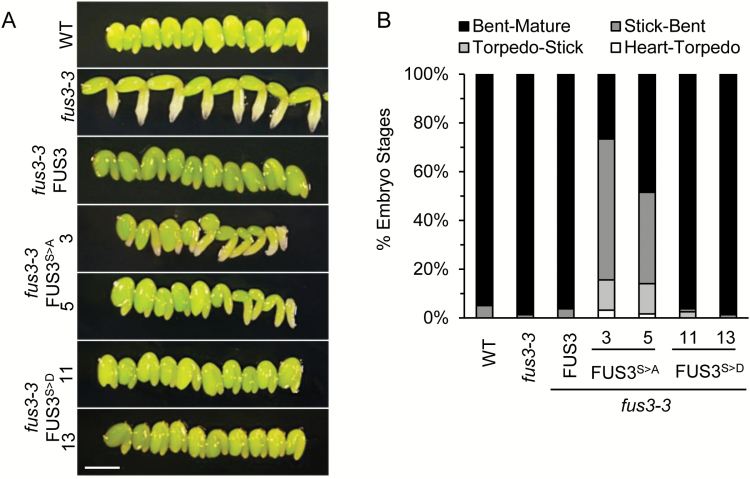
Delayed seed development of FUS3 phospho-null mutants at control temperature. Embryo development of wild-type, *fus3-3,* FUS3^S>A^, and FUS3^S>D^ plants grown under long days at 21 °C/18 °C. (A) Representative images showing the morphology of FUS3 phosphomutant embryos excised from 11 DAP siliques, corresponding to the bent cotyledon/mature stage in the wild type. *fus3-3* embryos skip dormancy and enter vegetative growth precociously, as seen from root growth and embryo size. FUS3^S>A^ embryo growth is delayed, as seen from the presence of early embryo stages (torpedo and walking stick). Scale bar=0.5 mm. (B) Quantification of embryo stages shown in (A). Embryos dissected from four siliques per genotype were scored for morphological stages (*n*=60–80). (This figure is available in colour at *JXB* online.)

Upon closer inspection, many FUS3^S>A^ siliques also showed delayed embryo development. While almost all wild-type embryos reached the late bent cotyledon stage at 11 DAP, <50% of the FUS3^S>A^ embryos were at a comparable stage (Fig. 4). Most FUS3^S>A^ embryos were closer to the walking stick stage and 10–15% were still at the torpedo or earlier stages. In contrast, the developmental progression of FUS3^S>D^ embryos was not significantly different from that of the wild type. Unlike FUS3^S>D^, FUS3^S>A^ did not rescue and instead enhanced the *fus3-3* cotyledon defects, such as polycotyledons (1.5%), previously reported (Table 3; Tsai and Gazzarrini, 2012*a*). Together, these results indicate that FUS3 phosphorylation promotes embryo growth and is required for proper cotyledon development.

**Table 3.** Frequency of cotyledon defects in FUS3 phosphomutants

Genotype	Total no. of seedlings	Defective cotyledons^*a*^	% Defective cotyledons
Wild type	1050	0	0%
*fus3-3, FUS3*	1050	2	0.2%
*fus3-3, FUS3* ^*S>A*^ *(3*)	1050	60	5.7%
*fus3-3, FUS3* ^*S>A*^ *(5*)	1050	46	4.4%
*fus3-3, FUS3* ^*S>D*^ *(11*)	1050	3	0.3%
*fus3-3, FUS3* ^*S>D*^ *(13*)	1050	2	0.2%

^*a*^ Cotyledon defects include altered cotyledon number (polycotyledons or single cotyledon), fused cotyledons, and twin embryos.

**Fig. 5.**
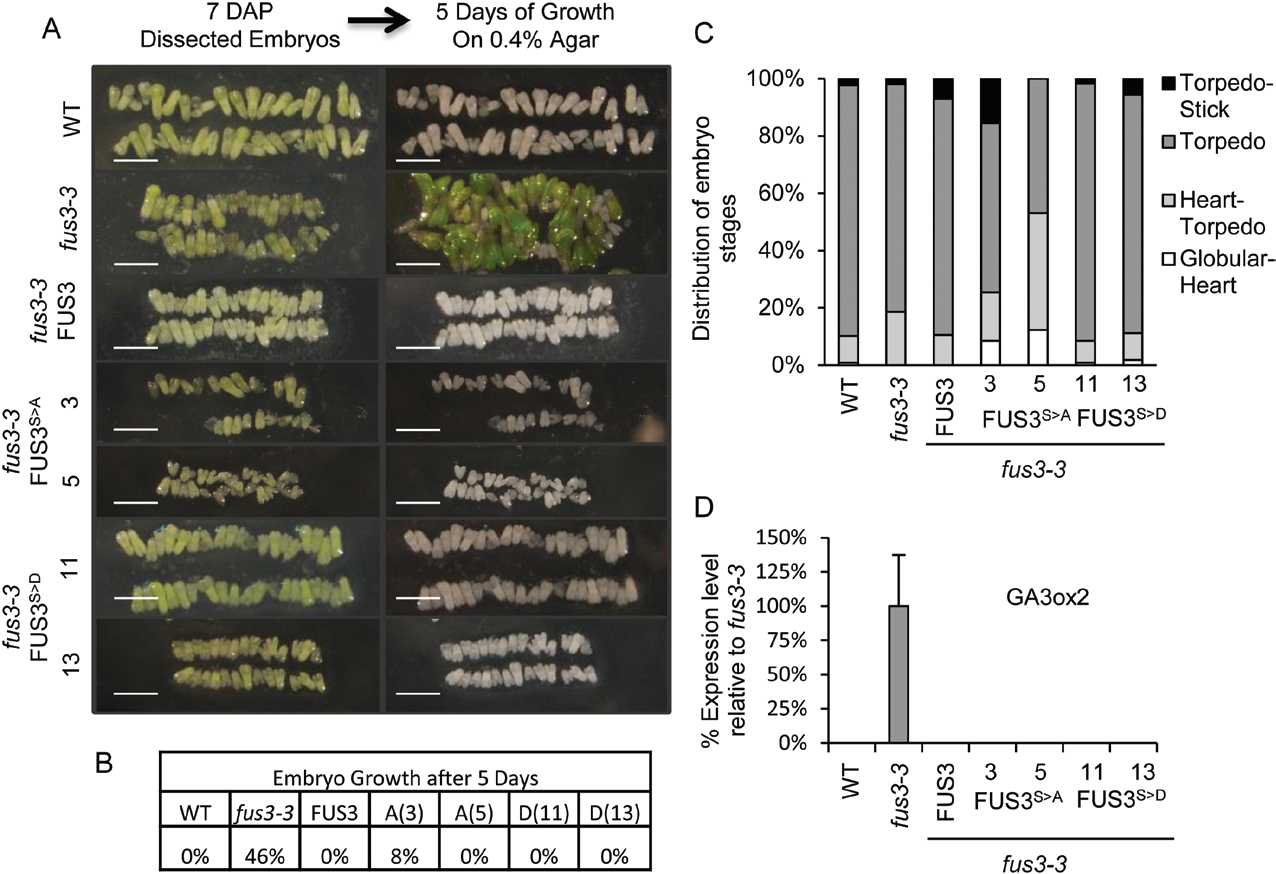
Suppression of growth in excised FUS3 phosphomutant embryos. (A) Representative images showing growth (*fus3-3*) or suppression of growth of embryos on 0.4% agar for 5 d. Arabidopsis plants were grown under long days at 21 °C/18 °C, and embryos at 7 DAP were dissected from four siliques (*n*=100–200). Scale bar=1 mm. (B) Quantification of excised embryo growth. (C) Distribution of total developing seeds excised from four siliques (7 DAP). (D) Transcript levels of *GA3ox2* measured by qPCR. *GA3ox2* expression was only detected in *fus3-3*. All plants were grown under long days at 21 °C/18 °C and siliques were collected at ~10 DAP (walking stick/bent cotyledon stages). (This figure is available in colour at *JXB* online.)

In agreement with the literature, *fus3-3* embryos showed precocious vegetative growth, as shown by growth of the radicle (Keith *et al.*, 1994), and all FUS3 phosphomutants rescued this phenotype (Fig. 4A). Previous work has shown that *fus3-3* mutants fail to suppress growth of excised embryos (Raz *et al.*, 2001). Therefore, we asked if the same growth suppression mechanism caused the difference in embryo development arrest or delay. Using the same growth assay on torpedo stage embryos (7 DAP), we observed no significant difference in the ability to complement the growth suppression defect of *fus3-3* by both FUS3 variants (Fig. 5A, B). Accordingly, expression of *GA3ox2*, a GA biosynthetic gene, is derepressed in *fus3-3* but not in the FUS3 phospho-mutant embryos (Fig. 5D; Curaba *et al.*, 2004; Gazzarrini *et al.*, 2004; Wang and Perry, 2013). A lower level of asynchronous embryo development was also observed in the early embryo stage of *fus3-3* (Fig. 5C). Overall, these data indicate that FUS3 phosphorylation positively regulates the embryo growth rate, but is not required for suppression of precocious germination.

### FUS3 phosphorylation is essential for plant growth and seed development at high temperature

**Fig. 6.**
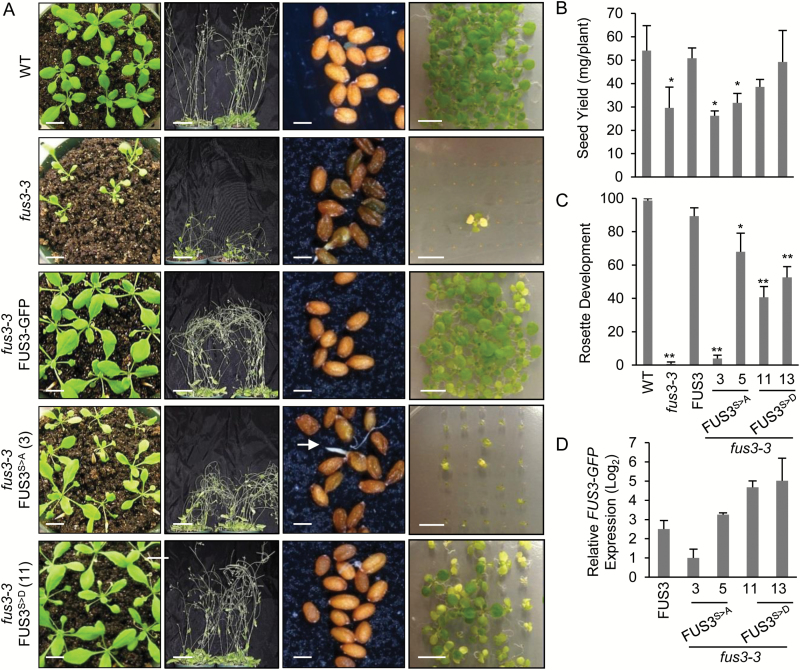
Vegetative and reproductive growth of FUS3 phosphomutants is greatly affected at elevated temperatures. Phenotypic analysis of FUS3 phosphomutants germinated for 7 d on agar plates and transferred to soil under long days at elevated temperatures (27 °C/24 °C). (A) Vegetative growth of *fus3-3* and FUS3^S>A^ mutants is greatly affected at high temperature, as can be seen from reduced rosette size (first column), less erect inflorescences (second column), and increased numbers of shriveled seeds with occasional vivipary (white root shown by arrow) (third column) and lowered seedling establishment of the progeny (fourth column). Scale bars=(left to right) 1, 5, 0.1, 0.9 cm. (B) Seed yield is greatly reduced in *fus3-3* and FUS3^S>A^ plants compared with the wild type. *n*=8 plants; *P*<0.05 (Student’s *t*-test). (C) The progeny of FUS3 phosphomutants show lower seedling establishment (emergence of leaf primordia) 15 d after imbibition. The averages of three plates of 50 seeds ±SD are shown. (D) Relative transcript levels were measured by qPCR. Siliques were collected approximately at the bent cotyledon stage (9 DAP). The FUS3^S>D^ transgene is expressed at higher levels compared with FUS3, while the FUS3^S>A^ transgene (line 3) shows a lower expression level. (This figure is available in colour at *JXB* online.)

**Fig. 7.**
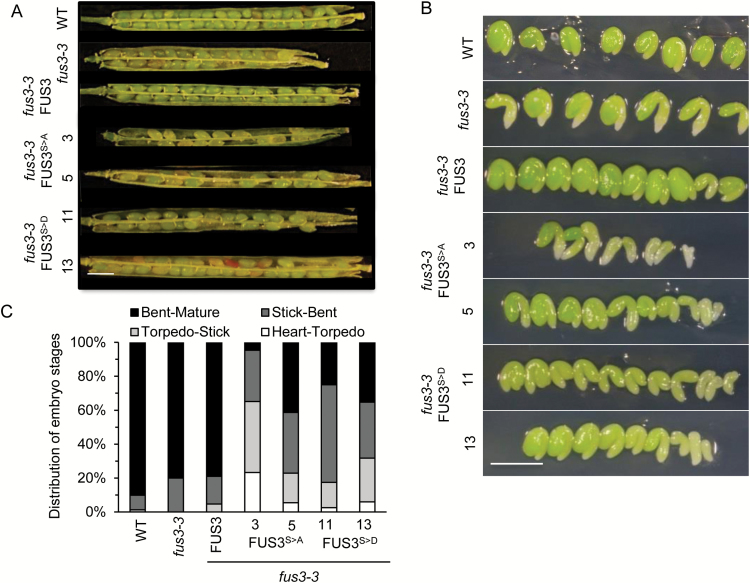
Development of FUS3 phosphomutant embryos at elevated temperature. Embryo development was examined in FUS3 phosphomutants grown under long days at elevated temperature (27 °C/24 °C). (A) Representative images showing aborted seeds in siliques at 14+ DAP. Scale bar=1 mm. (B) Representative images showing embryo morphology of FUS3 phosphomutants dissected from siliques at 9 DAP corresponding to the bent cotyledon/mature stage in the wild type. Scale bar=0.5 mm. (C) Quantification of embryo developmental stages in siliques at 9 DAP; four siliques per genotype (*n*=60–80). (This figure is available in colour at *JXB* online.)

The FUS3 protein is not detected post-embryonically under optimal growth conditions; however, it accumulates in seeds imbibed at supraoptimal temperature to inhibit germination (Chiu *et al.*, 2012). We examined whether FUS3 phosphorylation by SnRK1, a major regulator of the stress response, plays a role during growth at elevated temperature. When grown under long days at 27/24 °C, the overall growth was negatively impacted in *fus3-3* and FUS3^S>A^ plants, which showed smaller rosettes, weaker inflorescences that were unable to stand upright, and reduced seed yield compared with the wild type (Fig. 6A, B). FUS3^S>A^ line 3 also produced shriveled seeds with a low germination rate, poor progeny seedling establishment, and yellowing of the cotyledons at 27 °C, similar to *fus3-3* (Fig. 6A, C). The mRNA level of *FUS3*^*S>A*^ of line 3 was strongly reduced, explaining the lack of *fus3-3* rescue of this line at 27 °C (Fig. 6D). When plants were grown at elevated temperature, both FUS3^S>A^ and FUS3^S>D^ variants caused delayed embryo development and increased seed abortion compared with the wild type and *fus3-3* (Fig. 7; Table 4). This suggests that while aspartic acid may mimic phosphorylation under control conditions, effective similarity is reduced by elevated temperature. Alternatively, constitutive phosphorylation (S>D) may not be as effective as regulated phosphorylation. Altogether, these data indicate an essential role for FUS3 phosphorylation during growth at high temperature. Given that neither FUS3 nor its phosphomutant variants were detected during vegetative growth at high temperature (Supplementary Fig. S4), these results also suggest that FUS3 phosphorylation in the mother plant integrates temperature cues to regulate growth and survival of the progeny indirectly.

**Table 4.** Frequency of aborted seeds in various genotypes grown at high temperature

Genotype	Total no. of seeds	Total no. of aborted seeds	% Aborted seeds
Wild type	283	29	10%
*fus3-3*	202	33	16%
*fus3-3, FUS3*	254	20	8%
*fus3-3, FUS3* ^*S>A*^ *(3*)	220	71	32%
*fus3-3, FUS3* ^*S>A*^ *(5*)	263	85	32%
*fus3-3, FUS3* ^*S>D*^ *(11*)	280	75	27%
*fus3-3, FUS3* ^*S>D*^ *(13*)	283	61	22%

Total seeds produced from eight siliques including aborted seeds were counted.

Seed abortion included both fertilized and unfertilized ovules.

Plants were grown under long days at 27 °C/24 °C.

## Discussion

The LAFL transcription factors are master regulators of the seed maturation program. Through alteration of the transcriptome, they co-ordinate the accumulation of seed storage compounds, the establishment of dormancy, and acquisition of desiccation tolerance, while suppressing germination (Jia *et al.*, 2013; Sreenivasulu and Wobus 2013; Fatihi *et al.*, 2016). Compared with their role in transcription, there is less known about upstream regulators and modulators of their functions. Previously, we have shown that FUS3 interacts with and is phosphorylated by the SnRK1α1/AKIN10 kinase on three serine residues in the conserved B2 domain (Tsai and Gazzarrini, 2012*a*). However, the biological role of this post-translational modification was not clear. Here we found that AKIN10 is expressed in embryos, from the triangular to mature stages, and phosphorylates FUS3 during early to mid stages of embryogenesis. Using FUS3 phosphomutants at the SnRK1 target sites, we show that FUS3 phosphorylation plays a crucial role during embryogenesis, by regulating embryo growth rate and seed development under optimal and stress conditions. Impairment of FUS3 phosphorylation negatively affects FUS3 transcriptional activity, increases seed abortion, delays embryo development, and ultimately results in a large decrease in seed yield. The importance of FUS3 phosphorylation is more evident when plants are grown at high temperature, as even germination and vegetative growth of the next generation is greatly impacted. This suggests that SnRK1 and FUS3 may integrate endogenous signals (energy level) and environmental cues to regulate seed development and ensure survival of the next generation.

Previously, we showed that SnRK1 phosphorylation positively regulates FUS3, and that *FUS3* acts downstream of *AKIN10* in Arabidopsis (Tsai and Gazzarrini, 2012*a*). However, AKIN10 was previously found to be expressed only in the ovule, but not in the embryo where FUS3 mainly functions (Bitrián *et al.*, 2011). Here we show that AKIN10 is indeed expressed in the embryo, and that FUS3 and AKIN10 protein expression patterns partially overlap during embryogenesis. This is in agreement with the expression of the regulatory subunit SnRK1βγ in embryos (Bitrián *et al.*, 2011). Furthermore, FUS3 is phosphorylated by AKIN10 in cell extracts of early to mid embryos, thus their interaction is biologically relevant. In pea seeds, SnRK1α knockdown lines are impaired in maturation and show phenotypes similar to those displayed by *fus3* and *abi3* mutants, including precocious germination, reduced ABA and seed storage levels, and reduced desiccation tolerance (Radchuk *et al.*, 2006, 2010). This suggests that SnRK1 plays a prominent role during seed development and maturation, and probably acts through regulation of AFL functions.

To understand the role of FUS3 phosphorylation by SnRK1, we transformed *fus3-3* with *FUS3:FUS3*^*S>D*^-GFP phospho-mimic and *FUS3:FUS3*^*S>A*^-*GFP* phospho-null constructs. Our expression studies indicate that down-regulated gene transcription in *fus3-3* mutants is fully restored in FUS3^S>D^, but only partially restored in FUS3^S>A^ embryos, suggesting that FUS3 transcriptional activity is increased by phosphorylation. Furthermore, *FUS3*^*S>A*^ transgene expression was reduced while that of *FUS3*^*S>D*^ was increased compared with *FUS3*. This may be due to reduced FUS3^S>A^ and slightly increased FUS3^S>D^ ability, respectively, to regulate the FUS3 promoter positively, as previously shown for FUS3 (Parcy *et al.*, 1997; To *et al.*, 2006; Wang and Perry, 2013). However, FUS3 phosphorylation by SnRK1 did not appear to be required for seed maturation at normal temperature. The dramatic *fus3-3* phenotypes, including reduced dormancy, low levels of seed storage compounds, desiccation intolerance, and ectopic trichomes on the cotyledons (Bäumlein *et al.*, 1994; Keith *et al.*, 1994; Meinke *et al.*, 1994), were rescued by expressing either *FUS3*^*S>A*^ or *FUS3*^*S>D*^ transgenes. Altogether, these data suggest that SnRK1 phosphorylation positively regulates *FUS3* expression and transcriptional activity, but does not play a prominent role during seed maturation. It is possible that the full rescue of *fus3-3* seed maturation phenotypes in FUS3^S>A^ lines may be due to the action of other LAFL genes. Expression of *FUS3* and its target genes is induced by other B3 domain proteins through binding of the RY motif, which may compensate for reduced *FUS3*^*S>A*^ transcriptional activity (Jia *et al.*, 2013).

Upon closer inspection of siliques from plants grown under optimal temperatures, we found that ~8% of the *fus3-3* seeds were aborted and, surprisingly, FUS3^S>A^ lines showed an increase in the frequency of aborted seeds (~30%) due to a maternal FUS3^S>A^ effect. Furthermore, FUS3^S>A^ lines also showed delayed embryo development, with 10–15% of the seeds still at the heart to torpedo stages when all wild-type embryos already reached the mature stage. This resulted in a reduction of seed yield by ~50% in FUS3^S>A^ plamts. Thus, it appears that in FUS3^S>A^ siliques only half of the seeds were able to develop fully and reach the mature stage, and these embryos did not show maturation defects. Notably, *akin10* and to a lesser extent *akin11* mutants showed frequencies of seed abortion close to that of *fus3-3*, and similar phenotypes were observed during seed development of antisense *SnRK1α* lines (with 30–50% reduction in SnRK1 activity) in pea, where 10% seed abortion and delayed embryo development was also reported (Radchuk *et al.*, 2006). Interestingly, FUS3 phosphorylation affects the vegetative development and flowering time of the progeny, despite the lack of FUS3 protein expression post-embryonically, suggesting a link between seed development and flowering time. While FUS3^S>D^ rescued all *fus3-3* embryonic phenotypes, the FUS3^S>D^ progeny flowered later than the wild type at control temperature, similarly to ML1:FUS3 plants that overexpress FUS3 post-embryonically (Gazzarrini *et al.*, 2004). During seed development, FUS3^S>D^ may alter the regulation of flowering-related genes such as *FLC*, whose expression in the embryo was shown to be required for its repression of flowering (Sheldon *et al.*, 2008), or DELAY OF GERMINATION1 (DOG1), which controls dormancy and flowering time through miRNA 156/172 (Huo *et al.*, 2016). DOG1 and miRNA 156/172 are FUS3 targets based on ChIP studies (Wang and Perry, 2013).

When plants were grown at high temperature, plant growth was accelerated and seed yield was reduced in all genotypes. All phenotypes displayed by FUS3^S>A^ lines were enhanced when plants were grown at high temperature, in particular, both *fus3*-3 and FUS3^S>A^ plants were less vigorous and unable to stand upright, and the progeny seedlings grew poorly compared with the wild type. This indicates that phosphorylation of FUS3 by SnRK1 is critical during heat stress and affects the survival of the next generation. Possibly, specific transcriptional activities may be altered if phosphorylation of FUS3 changes the nature of ternary transcription complexes with LEC1/NF-YB, which increase the transcriptional activity of *ABI3/LEC2* on the *OLE1* promoter (Baud *et al.*, 2016). Alternatively, this may arise from novel protein–protein interactions. Thus, FUS3 phosphorylation in the mother plant integrates temperature cues to regulate growth and survival of the progeny. Interestingly, progeny dormancy is regulated by maternal temperature through activation of the florigen Flowering Locus T in siliques (Chen *et al.*, 2014; Penfield and MacGregor, 2017). It will be interesting to determine if maternal temperature affects both progeny dormancy and flowering time through similar pathways, thus passing environmental memory across generations to optimize plant growth.

The mechanism of how prevention of FUS3 phosphorylation causes seed abortion and delayed embryo development is not known. However, one possibility is that FUS3-mediated regulation of cell division is dependent on energy levels, which fluctuate in response to growth conditions and are signaled through SnRK1. FUS3 was previously shown to regulate cell division negatively; ectopic cell division was observed in the *fus3-3* embryo, while FUS3 overexpression represses cell cycling (Raz *et al.*, 2001; Gazzarrini *et al.*, 2004). FUS3 directly binds to the promoter of *cycD3;1*, and cycD activity suppressors *KIP-RELATED PROTEIN* (*KRP*) *4* and *6*, which have reduced expression in the *fus3-3* mutant (Yamamoto *et al.*, 2010; Wang and Perry, 2013). Higher order mutants of the *cyclin-D3* (*cycD3*) cell cycle regulator subfamily also display seed abortion and delayed embryo development phenotypes shown by FUS3^S>A^ lines (Collins *et al.*, 2012). Thus, FUS3 phosphorylation may control the rate of cell cycling to modulate the rate of embryo development and transition to maturation. Recently, a mutant affected in a novel nuclear-localized protein, *red1*, also displayed seed abortion, delayed embryo development, and desiccation intolerance phenotypes described in this study (Du and Wang, 2016). However, the function of this gene is currently unknown.

In conclusion, this work uncovered a novel and essential role for FUS3 phosphorylation by SnRK1 during early embryogenesis at optimal and especially at high temperatures. Lack of phosphorylation impairs FUS3 function and results in unfertilized ovules, aborted seeds, and delayed embryo growth, suggesting metabolic and signaling impairment. Seed abortion, reduced seed yield, and overall plant growth were further impacted when plants were grown at elevated temperature, suggesting that phosphorylation is essential for FUS3 function under stress, possibly integrating energy and stress levels through SnRK1 phosphorylation to ensure the survival of the next generation. Future work will focus on elucidating the molecular mechanisms underlying these processes.

## Supplementary data

Supplementary data are available at *JXB* online.

Table S1. List of primers used in this research.

Fig. S1. AKIN10 protein expression levels during seed development.

Fig. S2. In-gel kinase assays.

Fig. S3. FUS3–GFP and its phospho-variants are undetectable during early vegetative development at control temperature.

Fig. S4. FUS3–GFP and its phospho-variants are undetectable during early vegetative development under elevated temperature.

## Supplementary Material

Supplementary_Table_S1_Figures_S1_S4
