# Corrigendum: SnRK1 phosphorylation of FUSCA3 positively regulates embryogenesis, seed yield, and plant growth at high temperature in Arabidopsis

**DOI:** 10.1093/jxb/erx379

**Published:** 2017-11-13

**Authors:** Aaron Chan, Carina Carianopol, Allen Yi-Lun Tsai, Kresanth Varatharajah, Rex Shun Chiu, Sonia Gazzarrini

**Affiliations:** 1 Department of Biological Sciences, University of Toronto Scarborough, Toronto, ON Canada; 2 Department of Cell and Systems Biology, University of Toronto, Toronto, ON Canada

*Journal of Experimental Botany,* Vol. 68, No. 15 pp. 4219–4232, 2017 doi: 10.1093/jxb/erx233

The original published version of this article spelled an author’s name incorrectly. The correct name is as follows: Kresanth Varatharajah.

