# Supplementary material for: SnRK1 phosphorylation of FUSCA3 positively regulates embryogenesis, seed yield, and plant growth at high temperature in Arabidopsis
Source: J Exp Bot. 2017 Jul 22;68(15):4219–31. doi: 10.1093/jxb/erx233 (PMC5853833; doi:10.1093/jxb/erx233)
Supplement: Supplementary_Table_S1_Figures_S1_S4 [file erx233_suppl_supplementary_table_s1_figures_s1_s4.pdf]

Table S1. List of primers used in this study.

Gene ID	Forward Primer<br>Reverse Primer
**CRU2**<br>At1G03880	TCGAACCATCCCAAATCATC<br>TTAGACCCCTTCCGTGAACA
**SCT**<br>At1G47540	TCGTTTTGGCTATCTTTGCAG<br>CAACGGCATACTCCACCTTT
**2S3**<br>At4G27160	CGATGAGTTCGATTTCGAGG<br>GGTGGTCTGGAAGGGACATT
**KCS18**<br>AT4G34520	ACAAGGCATTTATGCTGGAGA<br>CGCTCTCATCGTCTTCTTGTT
**GA3ox2**<br>AT1G80340	CCACCACCTCAAATACTGTGAA<br>GGCTCTGTCTGGTTCTGGAC
**ACT7**<br>AT5G09810	TCACAGAGGCACCTCTTAACC<br>CCCTCGTAGATTGGCACAG
**FUS3-eGFP**	TTGGGCTCCGTTGAGAATATC<br>GCAGATGAACTTCAGGGTCAG

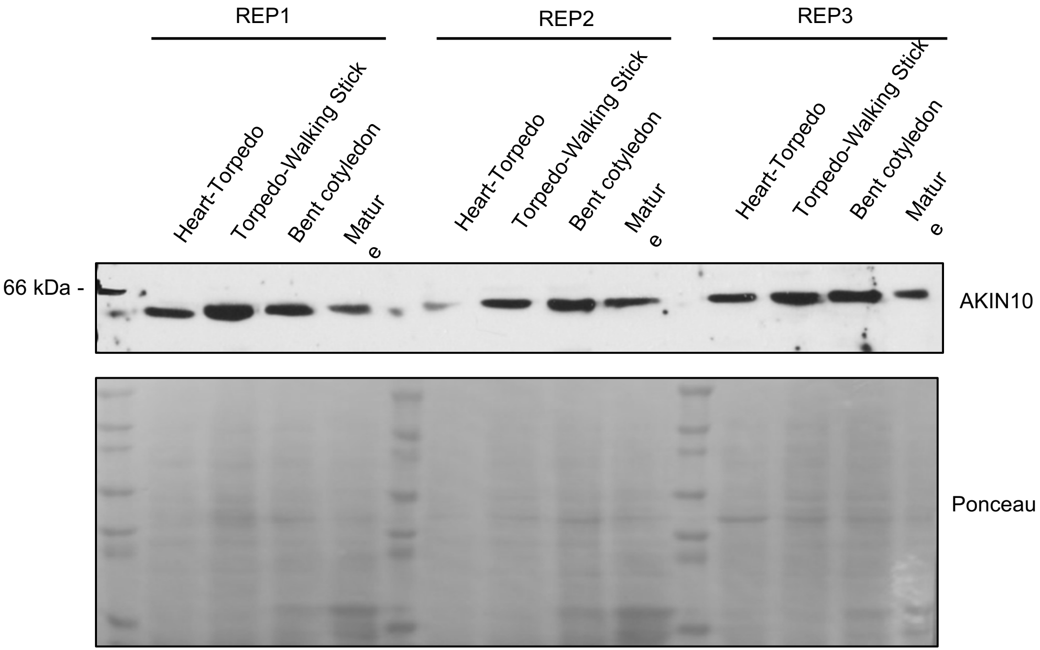

Supplemental Fig S1. AKIN10 protein levels during seed development

AKIN10 protein was detected throughout seed development from total soluble protein of isolated seeds using anti-AKIN10. Three biological replicates (REP) are shown. Ponceau stain is shown as the loading control.

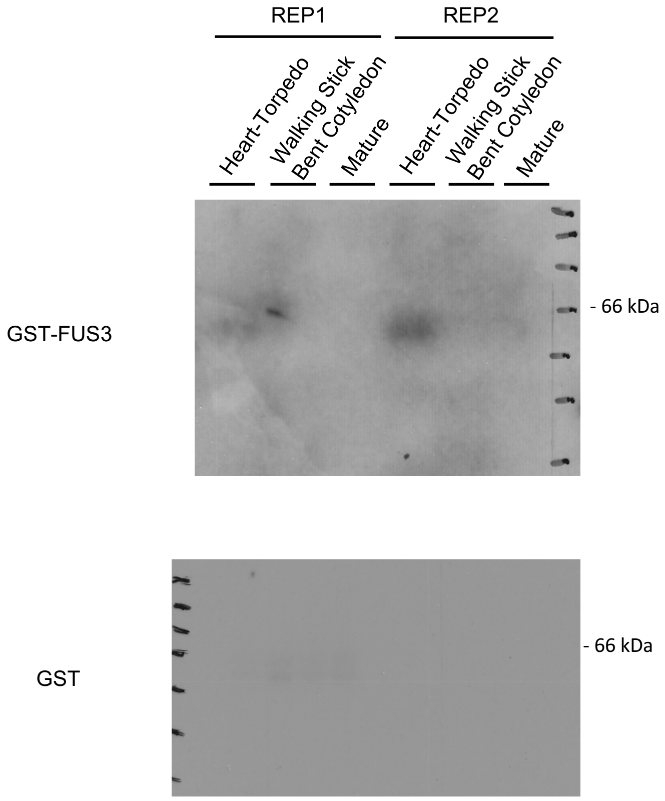

Supplemental Fig S2. In-gel kinase assays.

In-gel kinase assay using GST-FUS3 (top) or GST (bottom) as the substrates. Cell extracts from siliques corresponding to the the heart/torpedo stage embryos show a phosphorylation band at the expected size of AKIN10 (~60 kDa). Two biological replicates (REP) were conducted.

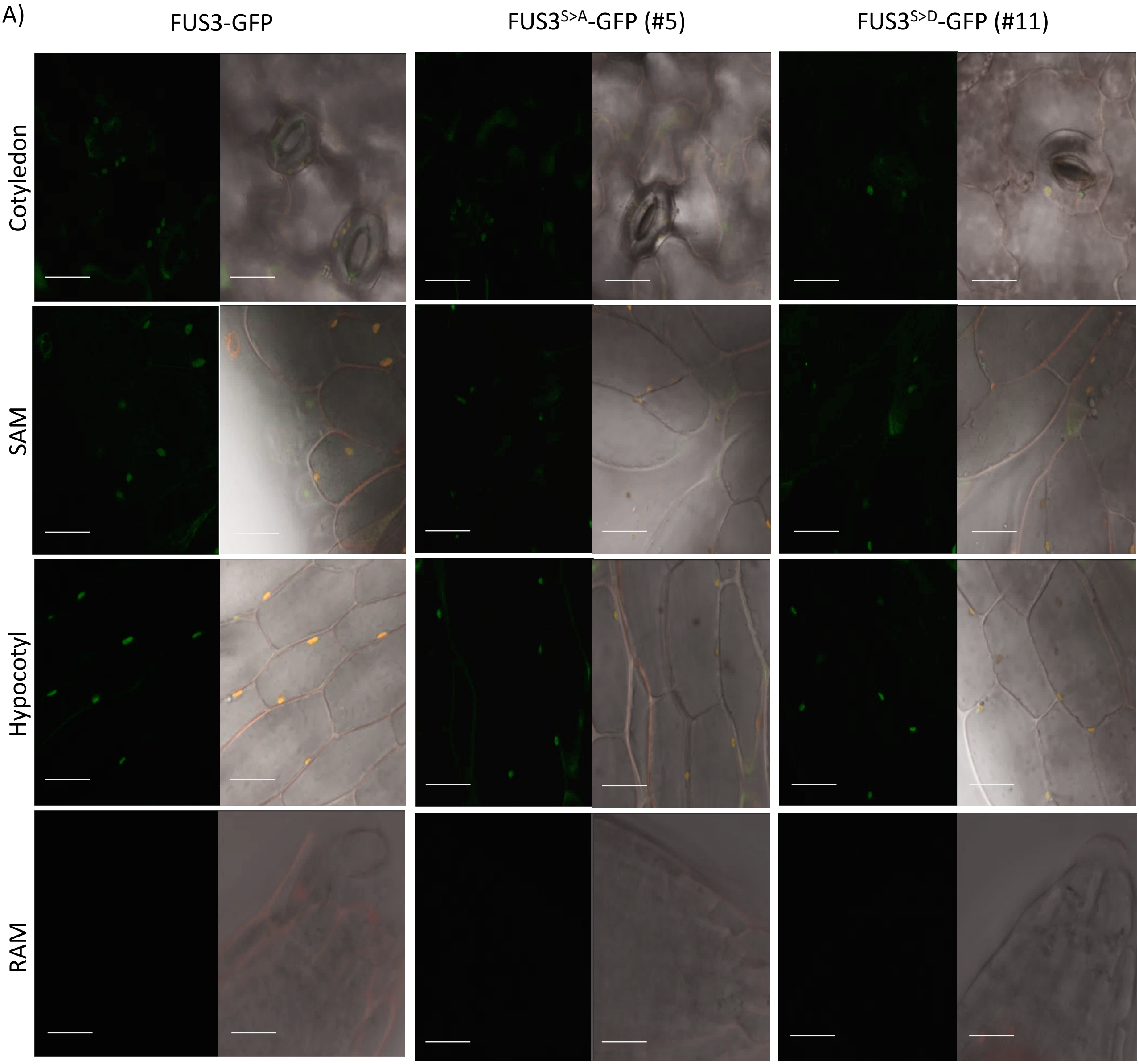
A)
FUS3-GFP
FUS3S>A-GFP (#5)
FUS3S>D-GFP (#11)
Cotyledon
SAM
Hypocotyl
RAM

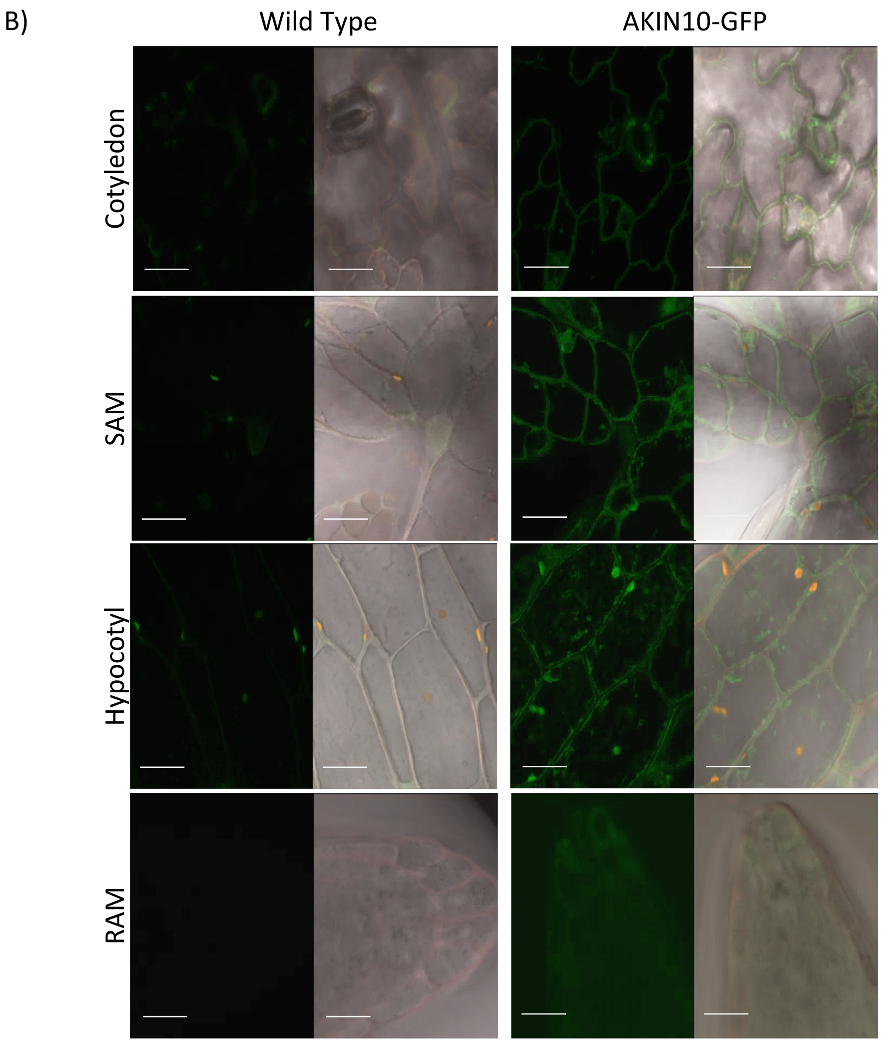

Supplemental Fig. S3. FUS3-GFP and its phospho-variants are undetectable during early vegetative development at control temperature.

A) Confocal images showing lack of expression of FUS3-GFP, FUS3-GFP(S>A) and FUS3-GFP(S>D) variants in the cotyledon, shoot apical meristem region (SAM), hypocotyl and root apical meristem region (RAM) of Arabidopsis 5-days-old seedlings grown on MS plates at control temperature. B) AKIN10-GFP was detected in all organs imaged. Similar results were obtained in 10-days-old seedlings. Plants were grown under long days at 21/18°C. Scale bars: 20 µm.

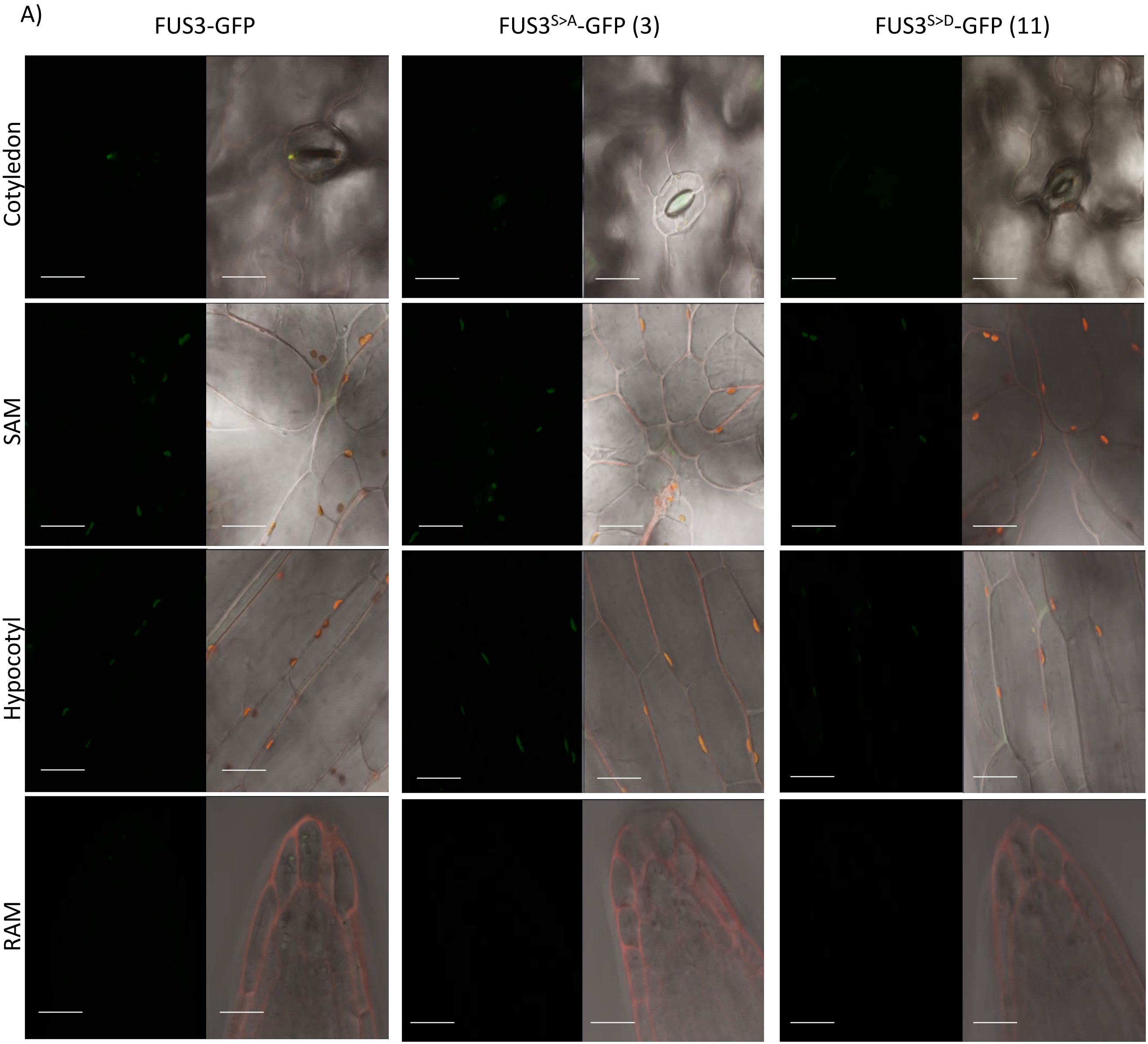
A)
FUS3-GFP
FUS3S>A-GFP (3)
FUS3S>D-GFP (11)
Cotyledon
SAM
Hypocotyl
RAM

B)

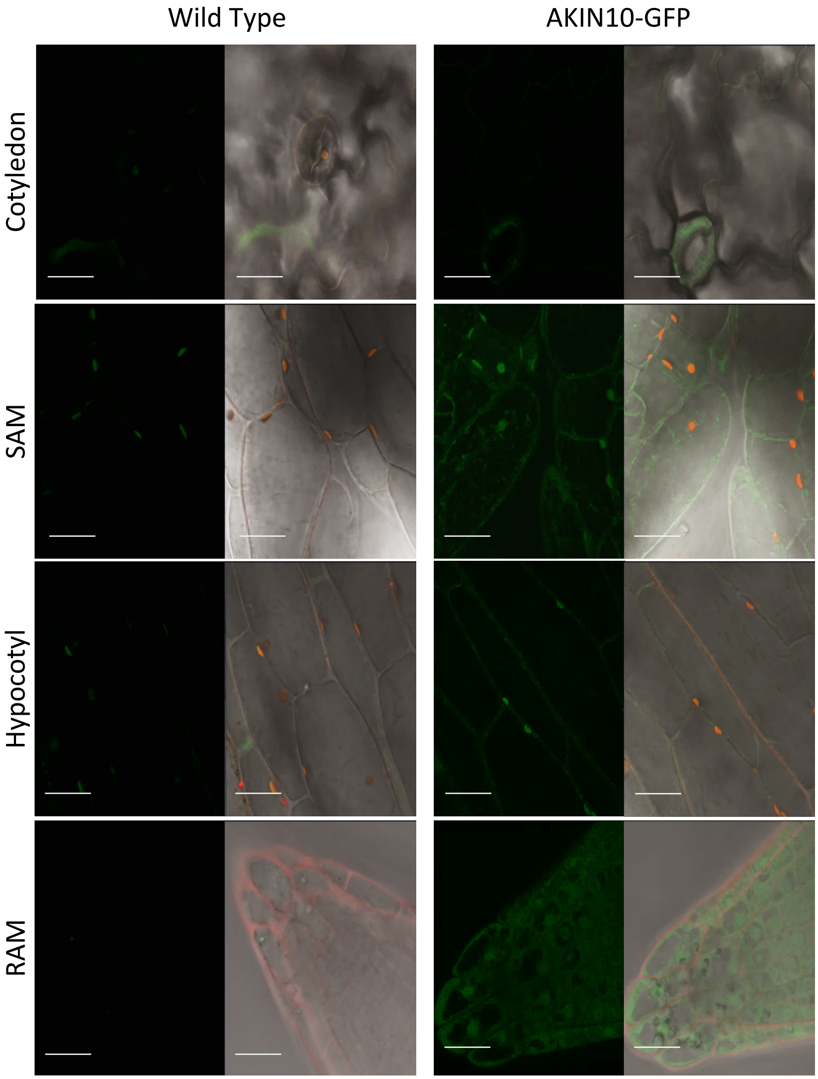

Supplemental Fig. S4. FUS3-GFP and its phospho-variants are undetectable during early vegetative development under elevated temperature

Confocal images showing lack of expression of FUS3-GFP, FUS3-GFP(S>A) and FUS3-GFP(S>D) variants in the cotyledon, shoot apical meristem (SAM), hypocotyl and root apical meristem (RAM) of Arabidopsis 5-days-old seedlings grown on MS plates at elevated temperature. AKIN10-GFP was detected in all organs imaged. Similar results were obtained in 10-days-old seedlings. Plants were grown under long days at 27/24°C. Scale bars: 20 µm.
